# Wealth- and education-related inequalities in minimum dietary diversity among Indonesian infants and young children: a decomposition analysis

**DOI:** 10.1080/16549716.2022.2040152

**Published:** 2022-04-07

**Authors:** Bunga A. Paramashanti, Michael J. Dibley, Ashraful Alam, Tanvir M. Huda

**Affiliations:** a Sydney School of Public Health, Faculty of Medicine and Health, The University of Sydney, Sydney, Australia; b Department of Nutrition, Faculty of Health Sciences, Universitas Alma Ata, Yogyakarta, Indonesia

**Keywords:** Minimum dietary diversity, socioeconomic disparities, concentration index, decomposition analysis, determinants, Indonesia

## Abstract

**Background:**

Over the last two decades, Indonesia has experienced remarkable economic growth. However, the percentage of infants and young children meeting the minimum dietary diversity (MDD) criteria has stagnated. Despite the growing body of evidence of the association between MDD and socioeconomic factors, there is little information about socioeconomic inequalities in MDD in Indonesia.

**Objectives:**

The current study seeks to quantify the wealth- and education-related inequalities in MDD among infants and young children in Indonesia and determine the contribution of different factors to these disparities.

**Methods:**

We included a total of 5038 children aged 6–23 months of the 2017 Indonesia Demographic and Health Survey. We measured wealth- and education-related inequalities using the concentration curve and Wagstaff normalised concentration index. Using a concentration index decomposition analysis, we then examined factors contributing to wealth- and education-related inequalities in MDD.

**Results:**

The concentration indices by household wealth and maternal education were 0.220 (p < 0.001) and 0.192 (p < 0.001), respectively, indicating more concentration of inequalities among the advantaged population. The decomposition analysis revealed that household wealth (29.8%), antenatal care (ANC) visits (16.6%), paternal occupation (15.1%), and maternal education (11.8%) explained the pro-rich inequalities in MDD in Indonesia. Maternal education (26.1%), household wealth (19.1%), ANC visits (14.9%), and paternal occupation (10.9%) made the most considerable contribution to education-related inequalities in MDD.

**Conclusions:**

There is substantial wealth- and education-related inequalities in MDD. Our findings suggest an urgent need to address the underlying causes of not reaching dietary diversity by promoting infant and young child feeding equity in Indonesia.

## Background

Malnutrition is a predominant public health issue among children. Globally, an estimated 22% or 149 million children under five are affected by stunting. Wasting remains to threaten the lives of an estimated 7% or 45 million children under five. Overweight affects an estimated 6% or 39 million children under five [1]. In Indonesia, child malnutrition rates remain alarming. The 2018 Indonesia Basic Health Research (Riskesdas), the most recent nationally representative survey, has reported a stunting prevalence at 31%, wasting at 10%, underweight at 18%, and overweight at 8% [2]. These rates indicate that Indonesia is making slow progress and are off track in meeting the Global Nutrition Targets [3].

Eating a variety of food in addition to breastmilk help infants and young children achieve optimum growth, health, and development [4,5]. A diversified diet also reflects the quality and quantity of food intake, food security, and micronutrient adequacy of children [6–8]. Children who consume a diversified diet are more likely to have a reduced risk of stunting [9–11]. Moreover, children from low- and middle-income countries (LMICs) could avoid more than 11 million stunting cases if 90% or more of infants and young children received food from different groups to meet the MDD criteria [12]. Minimum dietary diversity is also associated with a decreased risk of anaemia [13,14] and developmental delays [15,16]. Overall, MDD may have long term effects on adult human capital, health, and economic productivity [17].

The World Health Organization (WHO)/United Nations International Children’s Emergency Fund (UNICEF) has recommended infants and young children meet a minimum dietary diversity (MDD), consuming foods and beverages from at least five out of eight food groups during the previous day, starting from six months. These food groups include 1) grains, roots, tubers, 2) pulses, 3) vitamin A-rich fruits and vegetables, 4) other fruits and vegetables, 5) dairy products, 6) flesh foods, 7) eggs, and 8) breastmilk [4,18]. This food group method is a relatively simple and easy measurement used in survey settings [7]. Additionally, MDD has been used as one of the process indicators to monitor the effectiveness of various breastfeeding and complementary feeding interventions in the Global Nutrition Monitoring Framework [4].

Existing studies in LMICs has extensively examined factors affecting MDD at the child, maternal, household, and community levels. These studies have found that maternal education and household economic status are among the factors that are consistently associated with MDD. Children of higher economic status are at greater odds of receiving foods from diverse foods [19–23]. Mothers with higher education are more likely to have children who consume a more varied diet than those with lower education [19–21,23–25]. Other factors, such as maternal employment [26], paternal education [20,27], antenatal care (ANC) [20,25] and residency [21,26], are often related to increasing dietary diversity but have shown mixed results across studies. Overall, these findings suggest that socioeconomically disadvantaged children are more likely not to reach MDD.

Dietary diversity increases with economic improvements [12,28]; however, Indonesia may not be the case. Over the last 22 years, Indonesia experienced a substantial economic change. The poverty rate was halved from 24% in 1999 to 11.3% in 2004. The annual economic growth averaged 6% between 2005 and 2015 [29,30]. Yet, despite the overall economic improvement of the country, dietary diversity among children has stagnated. A nationally representative analysis study showed that the consumption of a minimum of five out of eight groups in Indonesia was 53.1% in 2007, 51.7% in 2012, and 53.7% in 2017 [23]. One important reason might be the persistent income inequality, which could have worsened the unequal access to nutrition, clean water, sanitation, and health services [29].

Socioeconomic inequalities pose a significant challenge to optimal feeding practices [28]. However, very few studies have examined the extent of socioeconomic disparities related to dietary diversity and the factors contributing to the inequality [31,32]. Moreover, no study has examined socioeconomic inequalities in dietary diversity in the Indonesian context. While earlier research has extensively estimated odds ratios to analyse the relationship between socioeconomic status and dietary diversity [19–25], the concentration index may better assess inequalities across the whole population (e.g. in a cumulative share of individuals ranked by household economic status). Furthermore, the concentration index can also be decomposed into a range of explanatory variables that influence socioeconomic-related inequalities [33]. Understanding socioeconomic inequalities in MDD may assist policymakers and public health professionals to target specific groups of the population at risk to improve child dietary diversity and reduce the burden of not meeting MDD on child well-being. Therefore, this paper aims to fill in the gaps in the existing literature by quantifying the extent of wealth- and educational-related inequalities in MDD and examining the contribution of explanatory variables to wealth- and educational-related inequalities among infants and young children in Indonesia.

## Methods

### Data source

We used data from the 2017 Indonesia Demographic and Health Survey (IDHS), nationally representative of the 34 provinces. Provinces are the largest subdivisions in Indonesia, followed by districts/municipalities, subdistricts, and urban/rural villages in the lower administrative units. The survey used a two-stage stratified sampling design. First, primary sampling units or census blocks (CB) were selected by probability proportional to size, where the size is the number of households listed in the 2010 population census. The CB was stratified by rural and urban areas with implicit stratification in each stratum by sorting the CB by the wealth index category. Second, 25 households were selected systematically from each CB. All women aged 15–49 were eligible for individual interviews in these households. The 2017 DHS report provides detailed information on the questionnaires and sampling procedures [34].

### Outcome variable

The study outcome, minimum dietary diversity (MDD), assesses the percentage of children 6–23 months of age who have consumed at least five out of eight food groups in the past 24 hours. The food groups include 1) grains, roots, and tubers; 2) legumes and nuts; 3) dairy products; 4) flesh foods; 5) eggs; 6) vitamin A-rich fruits and vegetables; 7) other fruits and vegetables; 8) breastmilk [18]. We coded the answers as either ‘1 = yes, consumed’ or ‘0 = no, not consumed’ [34].

### Socioeconomic status

We used two indicators of socioeconomic inequalities: household wealth and maternal education. The wealth index was computed based on household assets using principal component analysis, and the key household assets variables included ownership of infrastructures and amenities. Briefly, the principal component analysis estimates a cumulative wealth score for each household based on its asset [35]. We divided these scores into five quintiles, from the lowest 20% representing the poorest group to the highest 20% representing the richest group. We grouped maternal education into four categories: none or not completed primary school, completed primary school, completed secondary school, and completed higher education.

### Contributory factors to socioeconomic inequality in dietary diversity

We selected the contributory factors to socioeconomic inequality in dietary diversity based on our study on MDD determinants in Indonesia. For the present study analysis, we only included significant variables in relation to MDD found in our previous research [23]. These variables included child’s age (6–11 months, 12–17 months, 18–23 months), mother’s education (none or incomplete primary school, completed primary school, completed secondary school, completed higher education), mother’s access to media (none, at least one media), mother’s occupation (not working, agricultural, non-agricultural), father’s occupation (not working, agricultural, non-agricultural), number of ANC visits in the last pregnancy (<4 visits, ≥4 visits), household wealth (poorest, poorer, middle, richer, richest), area of residence (rural, urban), and regions (Java and Bali, Sumatera, Kalimantan, Sulawesi, Eastern Indonesia).

## Data analysis

To assess the socioeconomic inequality in dietary diversity, we calculated the concentration index [36], which is a widely used measure of socioeconomic inequality, and is written as:

$$C=\frac{2}{\mu }cov\left(h,\;r\right),$$

where *h* is the health variable in which inequality is measured, μ is its mean, cov denotes the covariance, and *r* is the individual’s fractional rank in the distribution of socioeconomic position [33]. The value of the concentration index ranges from −1 to +1. A negative value indicates a disproportionate concentration of MDD among the disadvantaged groups, whereas a positive value indicates a disproportionate concentration of MDD among the advantaged groups. Zero value means the absence of wealth- and education-related inequalities. However, our outcome is a binary variable, the bounds of the concentration index do not extend to −1 and +1, but equal to μ−1 and 1−μ. Therefore, we normalised the concentration index by dividing its value by its bound as proposed by Wagstaff et al. [37,38]:

$$C_{norm}=\frac{C}{1-\mu }$$

We also plotted the concentration curves to display the cumulative proportion of the MDD (y-axis) against the cumulative proportion of the children sorted by their household wealth and maternal education on the x-axis, beginning with the most disadvantaged and ending with the most advantaged groups. The curve that lies above the line of equality indicates that MDD is concentrated among the disadvantaged groups. Conversely, the curve below the equality line suggests that MDD is more concentrated among the advantaged groups. The farther the curve deviates from the line of equality, the greater the degree of inequality [33].

To ascertain the factors contributing to the observed socioeconomic inequalities in dietary diversity, we decomposed the concentration index to measure the explanatory variables’ contribution to wealth- and education-related inequalities in MDD. For a linear additive relationship between MDD (*y*) and a set of determinants $\left(X_{k}\right)$, such as

$$y=\;a+\;\sum_{k}\beta_{k}X_{k}+\varepsilon ,$$

allows the concentration index for *y* to be written as:

$$C=\sum_{k}\left(\beta_{k}{\overset{ˉ}{X}}_{k}/\mu \right)\;C_{k}+GC_{\varepsilon }/\mu ,$$

where μ is the mean of *y*, ${\overset{ˉ}{X}}_{k}$ is the mean of $X_{k}$, $C_{k}$ is the concentration index for $X_{k}$ (defined analogously to C), $\beta_{k}{\overset{ˉ}{X}}_{k}/\mu$ is the elasticity of MDD with explanatory variables, and $GC_{\varepsilon }/\mu$ is the generalised concentration index for the error term (ε). A negative contribution revealed that an independent variable operated towards the pro-poor distribution of MDD. In contrast, a positive contribution indicated that an independent variable worked towards the pro-rich distribution of MDD [33]. In this study, we applied Wagstaff’s correction [37,38] into the equation:

$$C_{norm}=\;\frac{\sum_{k}\left(\beta_{k}{\overset{ˉ}{X}}_{k}/\mu \right)C_{k}}{1-\mu }+\frac{GC_{\varepsilon }/\mu }{1-\mu }\;$$

As the outcome’s binary nature, we used a Generalised Linear Model (GLM) with a binomial family and probit link to decompose MDD inequality [39]. In addition, our analysis demonstrated the low level of multicollinearity with a mean of variance inflation factor (VIF) of 1.38. We also performed interaction tests among possible dependent variables (i.e. household wealth, maternal education, father occupation, ANC visits, residency, region), but statistically not significant. We used Stata version 17.0 (StataCorp, College Station, TX) for statistical analysis, with the significance level determined at *p* < 0.05. We applied the ‘svy’ commands throughout the analyses to adjust the survey design of the IDHS by including sampling weight, strata, and cluster.

## Results

### Characteristics of the study participants and prevalence of minimum dietary diversity

Table 1 presents the background characteristics of the study participants and the percentage of children who met the MDD criteria. We included a total of 5038 children aged 6–23 months in the analysis. The overall prevalence of MDD among children 6–23 months was 52.6% (95% CI: 45.6–49.2). The prevalence meeting standards for MDD was higher among children aged 18–23 months (62.7%) and those whose mothers and fathers attained at least a higher educational degree (66.5% and 66.7%, respectively). In addition, we found a wide gap in the proportion of MDD among children across different household wealth categories, with 39.9% in the lowest quintile and 55.3% in the highest quintile. The prevalence of MDD was exceptionally high among children who resided in urban areas (57.2%). Minimum dietary diversity also displayed a remarkable regional difference, ranging from 37.2% in Eastern Indonesia to 55.0% in Java and Bali.

**Table 1.** Characteristics of the study population and proportions of minimum dietary diversity (weighted n = 5038)

	Variable frequencies		Proportion of MDD		
Variables	n	% (95% CI)	n	% (95% CI)	*p*
Child factors					
Child’s age					<0.001
6–11 months	1639	32.5 (30.9–34.2)	553	33.7 (30.9–36.6)	
12–17 months	1785	35.4 (33.7–37.2)	1088	60.9 (57.7–64.1)	
18–23 months	1615	32.1 (30.4–33.7)	1012	62.7 (59.6–65.7)	
Maternal factors					
Mother’s education					<0.001
None or incompleted primary school	293	5.8 (5.0–6.7)	104	34.2 (28.2–40.8)	
Completed primary school	2340	46.5 (44.5–48.4)	1106	47.3 (44.6–50.0)	
Completed secondary school	1543	30.6 (29.0–32.3)	873	56.5 (53.5–59.5)	
Completed higher education	862	17.1 (15.7–18.6)	573	66.5 (62.7–70.0)	
Mother’s occupation					<0.001
Agricultural	357	7.1 (6.2–8.1)	130	36.3 (30.8–42.3)	
Non-agricultural	1876	37.3 (35.6–57.4)	1095	58.4 (55.6–61.0)	
Not working	2797	55.6 (53.8–57.4)	1422	50.8 (48.3–53.4)	
Mother’s access to media at least once a week					0.001
None	719	14.3 (13.1–15.6)	326	45.3 (40.8–49.9)	
Any media	4319	85.7 (84.4–86.9)	2326	53.9 (51.9–55.8)	
Paternal factors					
Father’s education					<0.001
None or incompleted primary school	332	6.7 (5.9–7.7)	134	40.3 (34.5–46.3)	
Completed primary school	2145	43.5 (41.5–45.5)	1044	48.7 (45.9–51.5)	
Completed secondary school	1724	35.0 (33.1–36.9)	943	54.7 (51.6–57.8)	
Completed higher education	729	14.8 (13.5–16.2)	487	66.7 (62.5–70.6)	
Father’s occupation					<0.001
Agricultural or not working	1080	21.9 (20.4–23.6)	458	42.4 (38.9–46.0)	
Non-agricultural	3841	78.1 (76.4–79.6)	2144	55.8 (53.8–57.9)	
Health care, household, and community factors					
Number of antenatal care visits					<0.001
<4	429	8.7 (7.7–9.8)	180	41.8 (36.6–47.2)	
≥4	4499	91.3 (90.2–92.3)	2452	54.5 (52.6–56.4)	
Household wealth					<0.001
Poorest	1010	20.1 (18.6–21.6)	404	39.9 (36.4–43.6)	
Poorer	1032	20.5 (19.0–22.0)	523	50.7 (47.0–54.3)	
Middle	1123	22.3 (20.8–23.9)	639	56.8 (53.2–60.4)	
Richer	995	19.7 (18.2–21.3)	602	60.5 (56.4–64.5)	
Richest	878	17.4 (15.9–19.2)	486	55.3 (51.0–59.5)	
Living residency					<0.001
Urban	2487	49.4 (47.7–51.0)	1422	57.2 (54.7–59.7)	
Rural	2551	50.6 (49.0–52.3)	1230	48.2 (45.6–50.8)	
Region					<0.001
Java and Bali	2850	56.5 (55.0–58.1)	1566	55.0 (52.2–57.7)	
Sumatera	1133	22.5 (21.2–23.8)	620	54.8 (51.3–58.1)	
Kalimantan	297	5.9 (5.4–6.5)	160	53.5 (48.0–59.1)	
Sulawesi	356	7.1 (6.5–7.7)	158	44.1 (39.5–48.7)	
Eastern Indonesia	402	8.0 (7.4–8.6)	150	37.2 (33.4–56.0)	

n and %: weighted count and proportion, respectively.

*p*: p-value based on the chi-square test.

### Socioeconomic inequality in minimum dietary diversity

**Figure 1.**
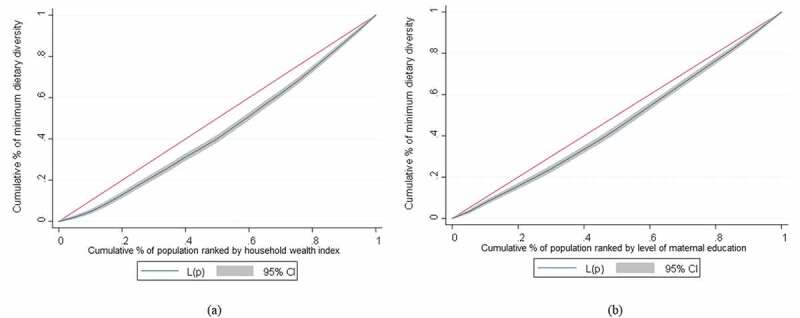
(a) Concentration curves of minimum dietary diversity ranked by household wealth index and (b) level of maternal education.

The normalised concentration indices (C_norm_) for MDD among infants and young children aged 6–23 months, ranked by household wealth and maternal education, are estimated at 0.220 and 0.192, respectively (see Table 2). The positive values of C_norm_ suggest that children from wealthier households and educated mothers had a more diverse meal. Figure 1 depicts the concentration curves for MDD among infants and young children aged 6–23 months, ranked by household wealth and maternal education. As illustrated, concentration curves lie below the 45-degree line, confirming that the proportion of MDD is higher in children with wealthier households and highly educated mothers.

**Table 2.** Wagstaff normalised concentration index of minimum dietary diversity by household wealth index and maternal education

	Wealth			Education		
	Index value	SE	*p*	Index value	SE	*P*
C	0.104	0.009	<0.001	0.091	0.009	<0.001
C_norm_	0.220	0.020	<0.001	0.192	0.019	<0.001

C: concentration index; C_norm_: Wagstaff normalized concentration index; SE: standard error; *p*: p-value.

### Contribution of the determinants to wealth- and education-related inequality in minimum dietary diversity

Table 3 summarizes the decomposition analysis results of wealth-and education-related inequality in MDD among children aged 6–23 months in Indonesia. Each column shows the elasticity of MDD, the concentration index, and the absolute and the percentage contributions of each contributor to the MDD concentration index. The elasticity shows how sensitive MDD is to each contributor. We found that MDD is mainly responsive to the child’s age, mother’s education, father’s occupation, and ANC visits.

**Table 3.** Decomposition of wealth- and education-related inequalities in minimum dietary diversity among Indonesian infants and young children

	Wealth				Education			
Variables	Elasticity	C_norm_	Absolute contribution	Relative contribution (%)	Elasticity	C_norm_	Absolute contribution	Relative contribution (%)
*Child factors*								
Child’s age								
6–11 months								
12–17 months	0.189	−0.018	−0.003	−1.4	0.189	−0.022	−0.004	−2.2
18–23 months	0.185	−0.006	−0.001	−0.4	0.185	−0.021	−0.004	−2.0
Subtotal		−0.024	−0.004	−1.9		−0.043	−0.008	−4.2
*Maternal factors*								
Mother’s education								
None or incomplete primary								
Completed primary	0.064	−0.413	−0.027	−11.2	0.064	−0.781	−0.050	−26.2
Completed secondary	0.076	0.256	0.019	8.2	0.076	0.507	0.039	20.0
Completed tertiary	0.062	0.566	0.035	14.8	0.062	0.997	0.062	32.3
Subtotal		0.408	0.028	11.8		0.722	0.050	26.1
Mother’s occupation								
Agricultural								
Non-agricultural	0.046	0.332	0.015	6.4	0.046	0.371	0.017	8.9
Not working	0.062	−0.151	−0.009	−3.9	0.062	−0.236	−0.015	−7.6
Subtotal		0.181	0.006	2.5		0.135	0.002	1.2
Mother’s access to media at least once a week								
None								
Any media	0.004	0.343	0.002	0.6	0.004	0.164	0.001	0.4
Subtotal		0.343	0.002	0.6		0.164	0.001	0.4
*Paternal factors*								
Father’s education								
None or incomplete primary								
Completed primary	−0.010	−0.425	0.004	1.7	−0.010	−0.496	0.005	2.5
Completed secondary	−0.023	0.263	−0.006	−2.5	−0.023	0.303	−0.007	−3.6
Completed tertiary	0.007	0.623	0.004	1.9	0.007	0.738	0.005	2.7
Subtotal		0.462	0.003	1.1		0.545	0.003	1.7
Father’s occupation								
Agricultural or not working								
Non-agricultural	0.069	0.521	0.036	15.1	0.069	0.305	0.021	10.9
Subtotal		0.521	0.036	15.1		0.305	0.021	10.9
*Health care, household, and community factors*								
Number of antenatal care visits								
<4								
≥4	0.093	0.426	0.039	16.6	0.093	0.308	0.029	14.9
Subtotal		0.391	0.036	16.6		0.308	0.029	14.9
Household wealth								
Poorest								
Poorer	0.014	−0.511	−0.007	−3.0	0.014	−0.238	−0.003	−1.8
Middle	0.033	−0.017	−0.001	−0.2	0.033	−0.050	−0.002	−0.9
Richer	0.036	0.500	0.018	7.5	0.036	0.195	0.007	3.6
Richest	0.061	1.000	0.061	25.7	0.061	0.568	0.035	18.1
Subtotal		0.972	0.071	29.8		0.476	0.037	19.1
Residency								
Urban								
Rural	0.008	−0.533	−0.004	−1.7	0.008	−0.290	−0.002	−1.1
Subtotal		−0.533	−0.004	−1.7		−0.290	−0.002	−1.1
Region								
Java and Bali								
Sumatera	0.017	−0.111	−0.002	−0.8	0.017	0.091	0.002	0.8
Kalimantan	0.005	−0.111	−0.001	−0.2	0.005	−0.066	0.000	−0.2
Sulawesi	−0.007	−0.271	0.002	0.8	−0.007	−0.033	0.000	0.1
Eastern Indonesia	−0.013	−0.563	0.007	3.1	−0.013	−0.065	0.001	0.4
Subtotal		−1.056	0.007	2.9		−0.072	0.002	1.2
Total			0.180	76.8			0.135	70.2
Residual			0.040	23.2			0.043	29.8

C_norm_: Wagstaff normalised concentration index.

The C_norm_ represents the degree of inequality in MDD for each contributor. As indicated by negative concentration indices, children of mothers with primary education (−0.413), fathers with primary education (−0.425), poorer households (−0.511), rural areas (−0.533), Sulawesi (−0.271) and Eastern Indonesia (−0.563) were highly concentrated among the poorer population. Similarly, children of mothers with primary education (−0.781), unemployed mothers (−0.236), fathers with primary education (−0.496), poorer households (−0.238), and rural areas (−0.290) were concentrated among the less educated population.

Table 3 shows the contributions of explanatory variables to wealth- and education-related inequalities in MDD. Mother’s education, father’s occupation, ANC visits, and household wealth explain most of the wealth- and education-related inequalities in MDD. The large elasticities of MDD for these contributors are responsible for their considerable contribution to MDD concentration indices. Conversely, there is a notable degree of wealth- and education-related inequalities in the father’s education and residency, but there is a minor sensitivity of MDD to variation in these contributors, thus making a small contribution to MDD concentration indices. Furthermore, considering that each contribution is the product of the sensitivity of MDD for that factor and the degree of wealth- and education-related inequalities in that factor, the positive or negative value of the contributor comes from the positive or negative elasticity or concentration index. For example, the contributions of being 12–17 months and 18–23 months old, having mothers with primary school, having unemployed mothers, having fathers with secondary school, belonging to poorer and middle economic status, residing in rural areas and residing in Kalimantan are negative. The negative contributions are derived from the negative elasticity or concentration index of these factors.

**Figure 2.**
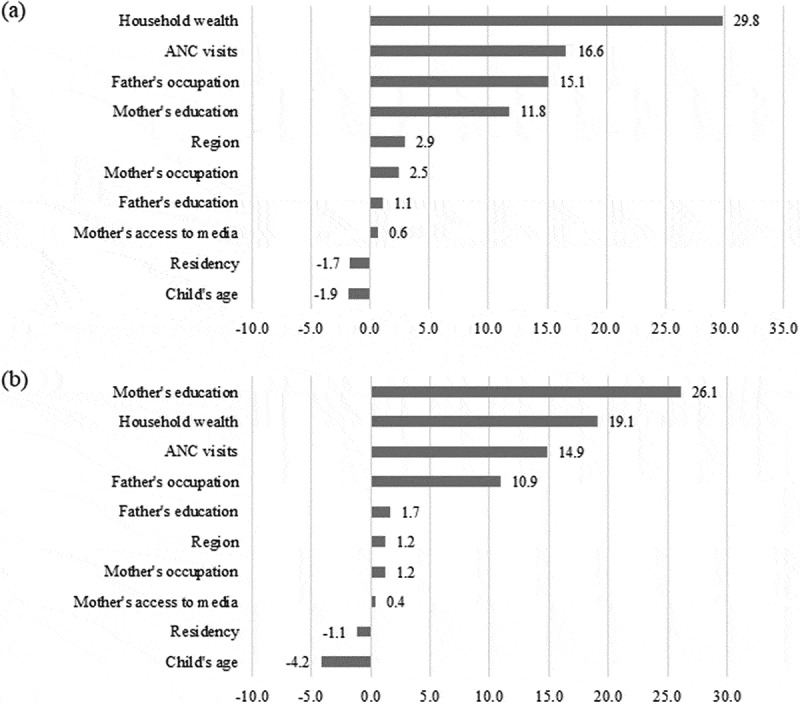
(a) Concentration curves of minimum dietary diversity ranked by household wealth index and (b) level of maternal education.

Figure 2 depicts the percentage contribution of the explanatory variables to wealth- and education-related inequalities. For wealth-related inequality in MDD, the largest contributor was household wealth (29.8%), followed by ANC visits (16.6%), paternal occupation (15.1%), and maternal education (11.8%). Similarly, the largest contributions toward education-related inequality in MDD included maternal education (26.1%), household wealth (19.1%), ANC visits (14.9%), and paternal occupation (10.9%). On the other hand, the child’s age, maternal employment, maternal access to media, paternal education, residency, and geographical regions showed minimal or no contribution to wealth- and education-related inequality in MDD. Overall, these variables explained nearly 76.8% and 70.2% of the wealth- and education-related inequalities in MDD.

## Discussion

This study is the first to examine the extent of wealth- and education-related inequalities in MDD among infants and young children and decomposed them into contributing factors in Indonesia. The study found that the proportion of children who had met the WHO’s minimum dietary diversity criteria was more concentrated among children from wealthier households and those born to mothers with higher educational attainment. Household wealth, mother’s education, father’s occupation, and ANC visits mainly contributed to the pro-rich and pro-educated socioeconomic inequalities in MDD.

Our result of pro-rich wealth-related inequalities in MDD was in line with previous studies [28,32]. Although no study has assessed education-related inequality in MDD, the distribution of infant and young child feeding indicators was higher among mothers with higher education in several studies [40,41]. However, these findings do not imply that eating a diversified diet does not occur among children from poorer families and less-educated mothers. Instead, it revealed MDD is disproportionately concentrated among the richer and educated population.

We found that household wealth was the predominant contributor to the wealth- (29.8%) and education-related (19.1%) inequalities in MDD. Similarly, earlier studies in Ethiopia [31] and Zimbabwe [32] have shown that household economic status was the main factor explaining socioeconomic disparities in MDD. Since dietary diversity is associated with the availability, access and utilisation of food, wealthier households are more likely to have enough resources to consume varied and nutritious food [42]. They have greater affordability to purchase non-staple foods, leading to improved dietary diversity [41,43]. At the same time, they also have better access to health care and information [44], thus applying the recommended feeding practices. Interventions that improve food purchasing power, such as income-generating strategies (e.g. homestead food production) and cash transfers, would help reduce the economic barriers to accessing a diversified diet [45–47]. In addition, infant and young child feeding promotions should be made available to all mothers and their children, especially those with lower economic status.

Of all the mother’s factors, maternal education is the most significant contributor to the wealth- (11.8%) and education-related (26.1%) inequalities in MDD. Although there has been no study examining the contribution of maternal education in the MDD inequalities, several studies have highlighted the contribution of this factor in explaining the disparities in child undernutrition [48–50]. The role of maternal education in improving child dietary diversity could be due to higher dietary knowledge [24,43,51] and better health literacy, dietary information-seeking behaviour, understanding, and critical thinking skills related to nutritional information [52]. Between 2002 and 2017, senior high school enrollment in Indonesia rose considerably from 50% to 71%. However, there was a 25% discrepancy in school enrollment between the poorest and wealthiest quintiles in the latest year. Moreover, 29% of these students did not complete their studies for various reasons, including insufficient funds, participation in the labour force, distance, marriage, and taking care of households for girls [53]. Thus, there is a need to narrow the gap in formal education participation across economic status, geographical regions, and gender [54], especially at the secondary and higher degree levels, for a long term investment in child nutrition. Governments should commit to encouraging school participation, for example, providing pro-poor incentives (e.g. cash transfers, food-for-education), decentralizing education to the district/municipality level, and developing alternative learning programs (e.g. non-formal education) [55]. Such initiatives should be designed to include people from marginalized communities, regardless of gender or ethnicity.

We also found that ANC visits had a distinct contribution to wealth- (16.6%) and education-related (14.9%) inequalities in MDD. Counselling received from the health practitioners during the visit, followed by appropriate practice, may lead to feeding children with a diversified diet [56]. In Indonesia, 96% of pregnant women had access to ANC services in 2018. However, only 74% met at least four ANC visits, ranging from 44% in Papua to 90% in Yogyakarta and 58% among women without formal education to 83% among women with a higher degree [2]. Increasing maternal awareness about ANC service by targeting the most vulnerable community is vital [50]. While the National Health Insurance (NHI) covers the ANC service fee, there is also a need to expand the NHI coverage to reduce sociodemographic inequalities in access to maternal and health services [57].

Our study also revealed that paternal occupation explained wealth- (15.1%) and education-related (10.9%) inequalities in MDD. Household head employment was associated with dietary diversity as it could determine the earnings [42]. However, Indonesian agriculture jobs dominated by small-scale farmers remain struggling with low incomes [58]. Such income disparities may increase the risk of food insecurity, making it difficult for them to afford healthy diets [59]. In addition to increasing crop production, a study in Bangladesh suggested that farmers could cover their household food expenses by seeking off-farm income. Thus, there is a need for policy support in agricultural development (e.g. best agronomic practices, access to information and credit, infrastructure investment) and off-farm income generating for smallholder farmers to achieve food security and lift them out of poverty [60]. In addition, there is much to learn from Tanzania, where nutrition-sensitive agriculture and agroecology interventions among food-insecure smallholder farmers have improved sustainable agricultural practices and women’ empowerment in income allocation, which could enhance household food security and children’s dietary diversity [61].

The development of nutrition education to improve a diversified diet in Indonesia began with the ‘Healthy Four Perfect Five’ (*Empat Sehat Lima Sempurna*) campaign. However, although this slogan encouraged people to eat various food groups (staples, plant- and animal-protein source food, fruits, vegetables), the value of milk as the ‘perfect’ food has been exaggerated. Milk mainly was not locally produced and costly, making it available only for the rich [62]. The most updated guideline, Guide for Balanced Nutrition, also encourages the population to eat a diversified diet by carrying a message of ‘be grateful and enjoy various food’. Nevertheless, this guideline is less socialized and implemented. Perhaps because of its simplicity, some industries and communities continue to use the old ‘Healthy Four Perfect Five’ [59]. Although the newest guideline has been developed for all populations across all ages, including children five years [63], some recommendations should follow the global indicators for infant and young child feeding practices, including minimum dietary diversity. Practical and straightforward messages may help communities adopt new nutritional information [64]. Health practitioners should adequately promote the nutrition guidelines by including locally available food [65] and pricing information [66] during all contact with mothers and young children, such as antenatal and postnatal care. Nutrition counselling and education should occur in multiple settings, involve local human resources, and reach out to mothers regardless of their socioeconomic backgrounds to ease disparities.

### Strengths and limitations

To our knowledge, this is the first study in Indonesia to measure both wealth- and education-related inequalities in MDD and to decompose the inequality by a set of contributing factors. The study used a nationally representative sample to generalise the findings to children aged 6–23 months in Indonesia. The use of the WHO’s most updated MDD indicator is helpful for ongoing monitoring and comparing with international guidelines [18]. However, MDD is constructed based on the single 24-hour food recall during the survey, thus not reflecting the actual feeding patterns [67]. While the decomposition analysis enables us to understand various factors contributing to the inequality in MDD, we could not draw a causal inference [33]. This issue also occurs when using cross-sectional data.

## Conclusions

The present study provided evidence on substantial wealth- and education-related inequalities in the MDD proportion among infants and young children in Indonesia. The overall findings of this study urge the need for multisectoral approaches to addressing the underlying causes of socioeconomic inequalities in MDD. We should prioritise children of poorer households and less educated mothers. Improving access and the quality of prenatal and postnatal health care is beneficial for delivering health-facility-based nutrition education. Nutrition-sensitive agriculture interventions may improve diet diversity through food production and income-generating. While there is a national recommendation on Balanced Nutrition Guideline, there is no evidence of whether the promotion of this guideline benefits infant and young child feeding and this issue requires further research. Finally, examining the changes of inequalities in MDD over time is vital for improving child nutrition outcomes in Indonesia.

## Paper context

While earlier studies have documented evidence on the determinants of minimum dietary diversity, very few have evaluated the magnitude and drivers of socioeconomic disparities in minimum dietary diversity. This paper provides evidence on wealth- and education-related inequalities in minimum dietary diversity among infants and young children in Indonesia. Our findings support calls for greater equality of wealth, education, and health care to close gaps in child nutrition.
